# Influence of different geographical factors on carbon sink functions in the Pearl River Delta

**DOI:** 10.1038/s41598-017-00158-z

**Published:** 2017-03-08

**Authors:** Qian Xu, Yuxiang Dong, Ren Yang

**Affiliations:** 1 Guangdong Provincial Key Laboratory of Urbanization and Geo-simulation, Centre of Land Research, School of Geography and Planning, Sun Yat-sen University, Guangzhou, 510275 PR China; 2 Xinhua College of Sun Yat-sen University, Guangzhou, 510520 PR China

## Abstract

This study analyzed carbon fixation across different land use types in the Pearl River Delta to identify the influence of different geographical factors on carbon fixation ability. The methodology was based on interpreting land use data from TM imagery, MODIS13Q1 data, and climate data, using the improved CASA and GeogDetector models. The results show that: (1) From 2000 to 2013, the total carbon sink increased slightly, from 15.58 × 10^6^ t to 17.52 × 10^6^ t, being spatially low at the center and increasing outwards; (2) Proxy variables (topography and landform characteristics), influencing urbanization, significantly affect the carbon sink function of the Pearl River Delta region. The proportion of urban and other construction land showed increasing effect on the regional carbon sink each year. However, the spatial structure of land in the study area changed from complex to simple, with enhanced stability; consequently, the influence of landscape characteristics (landscape dominance and landscape perimeter area fractal dimension) on the regional carbon sink gradually decreased; (3) The influence of the same factors differed with different land use types. Slope and altitude were found to have the greatest influence on the carbon sink of cultivated land, while landscape perimeter area fractal dimension more significantly affected the forest carbon sink.

## Introduction

This work clarifies the spatial and temporal evolution of carbon sink effects in urban systems and explores the influence of relevant geographical factors on their carbon fixation ability. Urban landscapes and their land use change characteristics are significantly different from those of natural ecosystems. The study of carbon dynamics in urban ecosystems and of the urbanization effect on carbon dynamics is still in its initial stages. The carbon stock of urban ecosystems, entering the carbon accumulation stage after functioning as a carbon sink, falls sharply, and there may be compensation before carbon loss, until a new, dynamic carbon balance is reached^1, 2^. There has been some research into factors influencing land use carbon sink effects within highly urbanized areas. The effect of carbon sinks in urban ecosystems has been examined from the perspectives of climate factor^3^ and climate change^4^, different land use types^5–12^ and land use change^13^, land use management^14^, transportation activities^15^, locational conditions^16^, the level of urbanization^1^, and so on. The urbanization process extensively influences the quantity, types, and spatial distribution patterns of land use, with the carbon sink function of different land uses changing accordingly. Some studies reported that forest land fluctuations in built-up areas represent the main contributors to carbon sink reduction and to increased carbon sources in urban systems^17^, and that urban areas have a lower carbon stock, with vegetation having lower soil organic carbon content than in rural areas^18^. Land use changes can influence the carbon cycles of terrestrial ecosystems via both carbon source and sink functions^19^. By changing the structure and function of regional ecological systems, land use change affects the ecosystem carbon cycle and its carbon absorption capabilities. Changes in land use have been widely regarded as a net carbon source of greenhouse gas emissions at the global scale, but in most studies, the main consideration has been the loss of carbon pool caused by the destruction of aboveground vegetation. However, Pugh *et al*. argued that increased atmospheric carbon dioxide could also increase the carbon sink in the terrestrial ecosystem^20^. There are clear differences in the carbon sink capacities of different land use types^21^, such that land use changes are often accompanied by significant carbon exchange^22^; this is one of the main influences on plant growth within land ecosystems and on soil carbon storage^23^. However, few previous studies have examined that land use structure, landscape form, and structural changes influence carbon sink. Previous studies predominantly assessed urbanization levels though the proportion of urban population; and less attention was given to the effect of land urbanization, because it was thought that the carbon sink function of urban ecosystems was mainly affected by the great increase in the scale of urban land rather than by the process of land use change.

The Pearl River Delta region was selected as the study area for this research. This is an important urban agglomeration area in China; it is densely populated and economically developed, with high levels of urbanization. We used remotely sensed land use data from the Landsat TM satellite (years 2000, 2005, and 2013), and applied CASA and GeogDetector models, taking into account climate, topography, landform, land urbanization, and landscape form and structure. We analyzed changes in land use carbon sinks within urban ecosystems, detecting the effects of geographical factors on the carbon sink function of the overall study area and of different land use types. We also identified the mechanisms by which various geographical factors and land use structures affect carbon sink functions.

## Results

### Spatial evolution characteristics of the land use carbon sink

**Figure 1:**
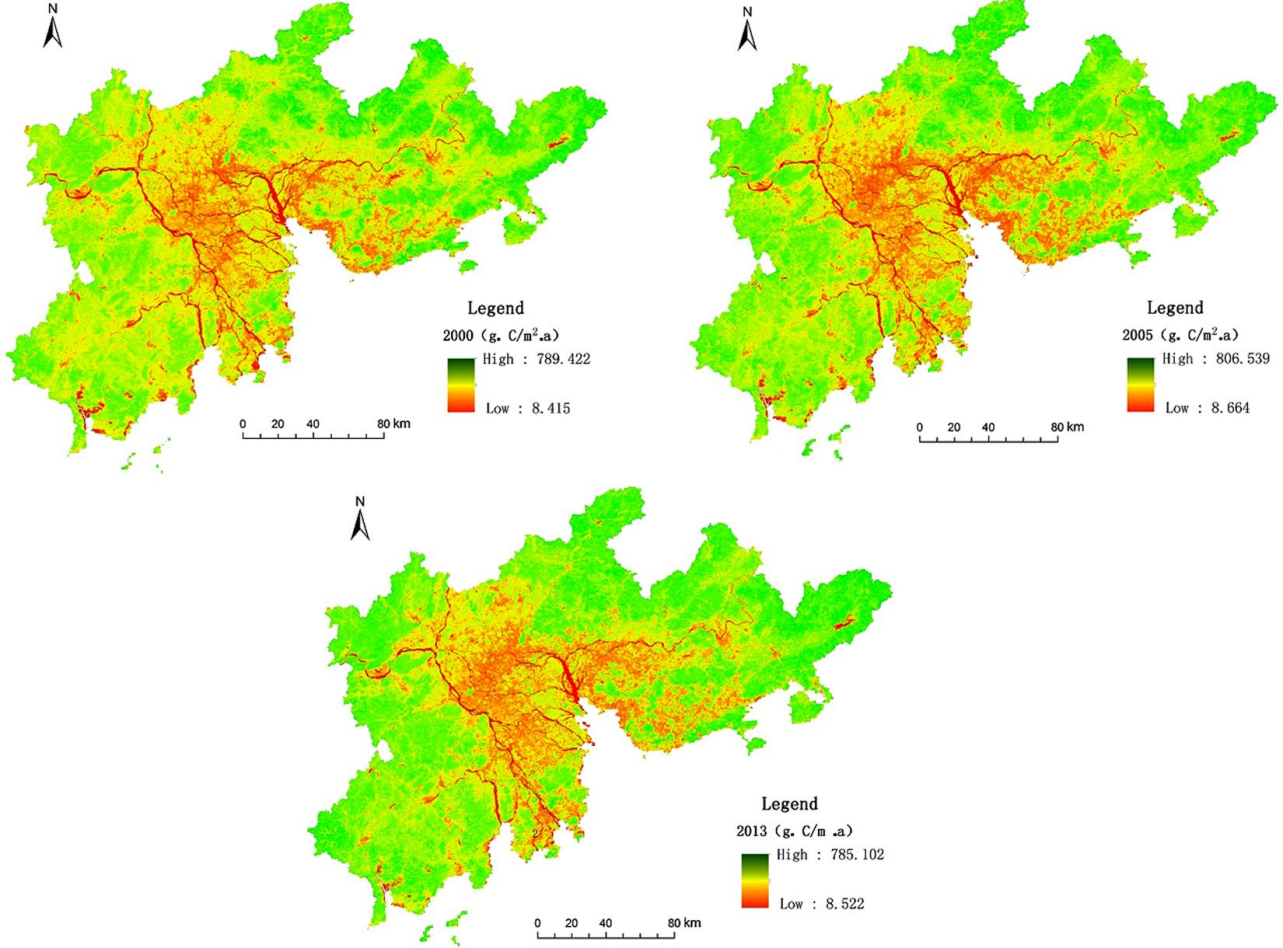
Spatial distribution of carbon sinks in the Pearl River Delta region. Map created using ArcMap (version 10.2) software from Esri (http://www.arcgis.com/).

The spatial distribution patterns of various land use carbon sinks display spatial differentiation in the amount of carbon fixed by vegetation. Moving outward from the Pearl River Estuary, there are clear increases in carbon fixation (low, medium, and high) across three concentric zones (Fig. 1). The carbon fixation capacity of different land use types also clearly differs, as follows: forest land > grassland > cultivated land > built-up > water area. Water areas are clearly low-carbon sinks, with the spatial pattern reflecting the low value distribution of the river network. The carbon fixation ability of lakes is slightly greater than of rivers but remains low. The central-south study area, near the Pearl River Estuary and the main urban zones of Guangzhou, Shenzhen, Foshan, and Dongguan, showed particularly and continuously low values. The extent of these low values increased from 2000 to 2005, with carbon fixation capacity per unit area also declining.

In terms of temporal evolution, from 2000 to 2013, the carbon fixation capacity per unit area of forest increased, following an initial decrease. In 2000, the carbon fixation values of forest was 520.14 gC·m^−2^·a^−1^, declined to 496.15 gC·m^−2^·a^−1^ in 2005, then increased in 2013 to 540.95 gC·m^−2^·a^−1^ (Table 1). The amount of carbon fixed by forests was significantly higher than that of other land use classes, measuring 10.12 × 10^6^ t in 2013. The carbon fixation capacity per unit area of grassland increased from 465.28 gC·m^−2^·a^−1^ in 2000 to 523.23gC·m^−2^·a^−1^ in 2013. Carbon fixation by grassland areas was low, totaling 0.38 × 10^6^ t in 2013. The carbon fixation capacity per unit area of cultivated land tended to increase year by year, with respective values of 418.69 gC·m^−2^·a^−1^, 430.08 gC·m^−2^·a^−1^, and 464.78 gC·m^−2^·a^−1^ in 2000, 2005, and 2013. Between 2000 and 2005 there was no evident change in the carbon fixation capacity per unit area of water, but this was the lowest of all land use types, with respective values of 278.98 gC·m^−2^·a^−1^ and 277.60 gC·m^−2^·a^−1^, increasing to 345.63 gC·m^−2^·a^−1^ in 2013. The fixation capacity per unit area of built-up land dropped from 299.64 gC·m^−2^·a^−1^ in 2000 to 296.61 gC·m^−2^·a^−1^ in 2005, but improved substantially in 2013 (342.81 gC·m^−2^a^−1^). From 2000 to 2013, the area of built-up land increased rapidly, with the total carbon sink doubling from 1.24 × 10^6^ t to 2.49 × 10^6^ t.

**Table 1:** Carbon values of different land use types in the Pearl River Delta area.

Type of land use	Carbon sink per unit area (gC·m^−2^·a^−1^)			Total carbon sink (×10^6^ t)		
	2000	2005	2013	2000	2005	2013
Cultivated land	418.69	430.08	464.78	5.29	4.86	4.95
Forest land	520.14	496.15	540.95	10.09	9.45	10.12
Grassland	465.28	485.95	523.23	0.39	0.38	0.38
Water area	278.98	277.60	345.63	1.17	1.13	1.33
Built-up land	299.64	296.61	342.81	1.24	1.79	2.49
Unutilized land	284.35	281.37	332.56	0.007	0.005	0.003

### Detection of geographic factors affecting the land use carbon sink

Using the GeogDetector model, we measured the impact of factors influencing the carbon sink in the Pearl River Delta area from 2000 to 2013. The key findings (see Table 2) are: (1) Topography and landform characteristics had obvious impacts on the regional carbon sink, but the influence of climatic factors was limited. Between 2000, 2005, and 2013, P values for the slope factor fluctuated but remained the highest of all impact factors (0.589, 0.578, and 0.603, respectively). P values for altitude were 0.426, 0.397, and 0.414, respectively. P values for annual mean temperature, annual rainfall, and annual mean humidity were significantly lower than those of other factors, although those of annual mean temperature in 2005 and annual mean humidity in 2013 were clearly higher than for other factors in the same year. (2) Land urbanization has a certain influence on the carbon sink function within the study area. Urban land proportion has a greater influence than other construction land, with both showing increasing influence year by year. In 2000, 2005, and 2013, P values for urban land proportion were 0.105, 0.150, and 0.207, compared with 0.089, 0.109, and 0.167 for other construction land; both sets of P values thus nearly doubled from 2000 to 2013. (3) Landscape ecological effects have significant influence on vegetation carbon sink capacity, but the influence wanes over time. In 2000, 2005, and 2013, P values were 0.282, 0.228, and 0.165, respectively for the Landscape Diversity Index; 0.281, 0.222, and 0.160 for the Landscape Dominance Index; and 0.112, 0.103, and 0.067 for the Landscape Perimeter Area Fractal Dimension. Landscape Diversity and Landscape Dominance have greater influence on the carbon sink than does the Landscape Perimeter Area Fractal Dimension.

**Table 2:** Geographically determined weightings of factors affecting carbon sinks.

Year	f_1_ (°C)	f_2_ (mm)	f_3_ (%)	f_4_ (m)	f_5_ (°)	f_6_ (%)	f_7_ (%)	f_8_	f_9_	f_10_
P value in 2000	0.084	0.033	0.014	0.426	0.589	0.105	0.089	0.282	0.281	0.112
P value in 2005	0.209	0.018	0.010	0.397	0.578	0.150	0.109	0.228	0.222	0.103
P value in 2013	0.044	0.015	0.084	0.414	0.603	0.207	0.167	0.165	0.160	0.067

The land use types with high carbon sink capabilities, such as forest land, grassland, and cultivated land, help determine the impact of various factors on different land use types, as shown for the year 2013 in Table 3. Here, since the present study considers only those factors that influence one kind of land use type with carbon sink capabilities, factors such as f_6_ (proportion of urban land proportion), f_7_ (proportion of other construction land), and f_8_ (Landscape Diversity Index) do not have any significance for detection and are therefore not considered. After the model was run, the following findings were obtained: (1) Among the forest, grassland, cultivated, and built-up land classes, the topography and landform factors are most influential, followed by landscape structure and form, with climate factors having minimal influence. (2) The factors have differential effects according to land use types across the overall study area. There are obvious differences in rainfall and humidity factors, between carbon sinks for single land use types and that of the overall study area. However, the impact of humidity on carbon sinks in forest, grassland, cultivated land, and built-up land areas is more evident than that of rainfall. (3) Impact factors affecting the carbon sink of different land use types have differentiated characteristics. Temperature and rainfall conditions have strongest influence on the carbon sink of built-up land. Humidity most strongly influences forest land and cultivated land. Altitude and slope have the strongest impact on the carbon sink of cultivated land, followed by forest land, and the influence of slope is more significant than that of altitude. When grass and forest occupy a greater proportion of a grid unit and the landscape dominance index is higher, the carbon sink function is stronger. The degree of landscape fragmentation has much more significant effect on forest land carbon sinks than on other land use types.

**Table 3:** Geographically determined weightings of factors influencing carbon sinks according to land use type (L_1_ forest; L_2_ grassland; L_3_ cultivated land; L_4_ built-up land).

Land use types	f_1_	f_2_	f_3_	f_4_	f_5_	f_9_	f_10_
P value of L_1_	0.041	7 × 10^−5^	0.064	0.373	0.573	0.241	0.138
P value of L_2_	0.028	0.002	0.008	0.293	0.476	0.308	0.067
P value of L_3_	0.041	0.010	0.088	0.382	0.586	0.171	0.093
P value of L_4_	0.056	0.018	0.057	0.254	0.463	0.012	0.002

## Discussion

### Causes of land use carbon sink changes

From 2000 to 2013, the carbon sink effect in the study area shows a “high middle and low around” spatial distribution. This is related to the spatial distribution of land use structure; the ecological protection zone located around the Pearl River Delta, where forest coverage is high; and very high proportion of construction land within the urban core. In terms of temporal evolution, the carbon sink values were 454.81 gC·m^−2^·a^−1^, 448.50 gC·m^−2^·a^−1^, and 478.79 gC·m^−2^·a^−1^ in 2000, 2005 and 2013, respectively (those value of carbon sink per unit area have been calculated according to Fig. 1 using ArcGIS 10.2 software). It is worth noting that the carbon sink capacity per unit area falls in the Pearl River Delta region from 2000 to 2005, but increases suddenly in 2013. This jump in the carbon sequestration value is remarkable because of high level of urbanization, frequent human activities, obvious non-agriculturalization of farmland, and rapid expansion of construction land area in the Pearl River Delta region. The reasons for this increase were considered to be related to either good climatic conditions in 2013 or an improvement in the ecological environment. Meanwhile, the carbon sink per unit area were evaluated at 448.50 gC·m^−2^·a^−1^, 409.52 gC·m^−2^·a^−1^, and 394.28 gC·m^−2^·a^−1^ in 2005, 2011, and 2012, respectively. However, measures intended to achieve qualitative improvement in the ecological environment are not realized in the space of one year (2012–2013); the high carbon sink value in 2013 was thus not a result of human activities. Further, the atmosphere data for Guangzhou, a central city in the Pearl River Delta, show that the minimum average daily temperatures can reach the destruction temperature levels and damage the vegetation^24^. Destruction temperature is an important meteorological index that influences vegetation growth. The minimum average daily temperatures in Guangzhou were 2.6 °C, 2.5 °C, and 2.5 °C, and precipitation was 1632.3 mm, 1813.9 mm and 2095.4 mm, respectively, in 2011, 2012, and 2013. These data show that compared to other years, the extreme minimum average daily temperature is higher and precipitation is more plentiful in 2013, which in turn suggests that the higher carbon sink value per unit area in 2013 is associated with climate condition, and has little to do with human activities.

Among the various land use types, the capacity for carbon fixation and the quantity of carbon fixed were the highest in forest land, in accordance with the results of previous studies^25, 26^. It is thus important to protect forests within the Pearl River Delta region; these are of significance not only for ecological conservation but also for increasing the vegetation carbon sink. Carbon fixation capacity per unit area of cultivated land has tended to increase year by year, but the area of cultivated land area has gradually declined. Between 2000 and 2005, 1092.09 km^2^ of cultivated land was converted to built-up area, and a further 920.62 km^2^ was converted between 2005 and 2013. The previous carbon sink within cultivated land has thus decreased. In water areas, carbon sequestration is mainly achieved through aquatic plant photosynthesis; the carbon dissolution activity of water itself is weak, and water areas thus have low carbon sequestration capacity. As noted, the Pearl River Delta contains urban agglomerations with high and rapid levels of urbanization; as a result, forest land, grassland, and cultivated land are being converted to built-up land, i.e., vegetated surfaces are being rendered impervious, with destruction of the ground vegetation layer, consequent loss of carbon sequestration capacity, and significant decline in the quantity of carbon fixed. However, urban planning has also resulted in the establishment of greenbelts and city parks. Furthermore, forest land management in urban areas can achieve better fixation performance than natural vegetation areas^11, 14^, and the ability of such land to act as a carbon sink has therefore increased after a period, subsequently achieving a new carbon balance. By 2013, the total carbon fixed by ecosystems had increased. On the one hand, this was due to good weather conditions, including high humidity that is suitable for vegetation growth and that enables greater accumulation of plant biomass.

### Impacts of geographical factors and land use structure on carbon sink effects

Based on high-resolution MODIS NDVI data and the improved CASA model, we evaluate the temporal evolution and spatial distribution patterns of land use carbon sink effects in the Pearl River Delta region of rapid urbanization. Compared to methods that utilize statistical data and empirical parameters, our methodology provides more accurate assessments and more intuitive spatial distribution pattern. Based on the two main aspects of land urbanization and landscape ecological pattern, the influence of urbanization on land use structure is demonstrated, and the effect of land use structure on carbon sinks during the process of urbanization is analyzed. Through this study, the factors associated with rapid urbanization process, especially land use structure, that affect the carbon sink capacity of typical urban agglomerations were identified.

**Figure 2:**
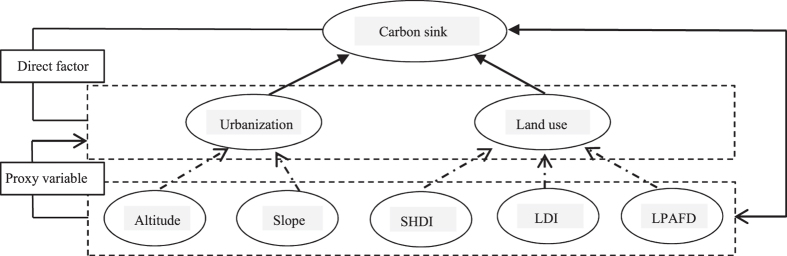
Proxy variable diagram (SHDI Shannon Landscape Diversity Index; LDI Landscape Dominance Index; LPAFD Landscape Perimeter Area Fractal Dimension).

Of the natural factors considered, altitude and slope factors were identified as limiting/driving factors of urbanization. Landscape ecological indices provide insight into the characteristics of land use change during the process of urbanization, but do not directly represent impacts on the carbon sink; however, they illustrate indirect effects through transformation associated with urbanization and land use changes, and can thus be classified as proxy variables^27^ (Fig. 2).

The Pearl River Delta region is an alluvial plain, with generally flat terrain; high elevation areas are mainly limited to important forest and nature reserves, including: Neilingding Island - Futian nature reserve on the east side of LingDingYang, Luofu mountain, Xiangtou mountain in Boluo county (Huizhou city), and Dinghu mountain (Zhaoqing city). Under different slope conditions, soil water and nutrient retention functions also differ, thus significantly affecting the carbon fixation ability of terrestrial ecosystems. The influences of slope and altitude factors are significantly greater for cultivated land than other land use types. Sloping cultivated land (slope of geomorphic types between 6° and 25°) greatly restricts improvement of crop yield. China’s policy of “returning farmland to forest and grassland” explicitly stipulates that cultivated land with slope >15° must be returned to forest or grassland. The impact of humidity factors on high-carbon sink types (forest land, grassland, cultivated land) is more important than that of rainfall conditions. Study results indeed show that climatic conditions have low P value; however, one possible reason is the short time-scale of research; as such, climatic conditions may still affect the regional geographical environment over the long term. In addition, there was no consideration of the vertical zonality law applicable to mountains, which experience the mutual influence of different natural geographical factors, including climate, hydrology, topography, and so on. Temperature, rainfall, and humidity conditions at different heights clearly differ; however, the temperature factor used in this study is the average temperature of the Earth’s surface.

As noted, the influence of the proportion of urban and other construction land has increased, and the expansion of built-up land (urbanization) has an obvious impact on vegetation carbon fixation, especially when a significant area of cultivated land is converted to built-up area. The original carbon balance of the regional ecological system is altered by the change in surface coverage. However, with increasing improvement of city functions and with the continuous development of urban form, the spatial structure of urban land uses in the Pearl River Delta region has tended to become more complex^22^, and the carbon cycle of the city system is tending towards a new equilibrium model.

In urban agglomerations cantered on cities, frequent human activities have greatly influenced the dynamic character of landscape form, then affecting landscape structure. To a certain extent, landscape structure determines landscape function, including the ecosystem carbon sink function. Landscape Diversity, Landscape Dominance, Landscape Perimeter Area Fractal Dimension, and other landscape ecological indices show an evident impact on the carbon sink of the Pearl River Delta. This indicates that landscape heterogeneity, the equilibrium distribution of different patch types in the regional environment (showing diversity changes of different or the same landscape over different periods), the degree of land use, the degree of patch fragmentation, the complexity characteristics of landscape spatial structure, and other such factors all influence the carbon sink function of terrestrial ecosystems to different degrees. However, with increasing urbanization, the scale of urban land within the Pearl River Delta region has grown and the spatial structure of land use has changed from complex to simple, enhancing stability^28^. The influences of landscape form and structure on the regional carbon sink function have thus gradually declined.

### Uncertainties

At present, there is agreement among the scientific community that terrestrial ecosystems provide a very large carbon sink function; however, there is still great uncertainty regarding the size and spatial distribution of this carbon sink^29^. For example, Mukul *et al*. argued that tropical secondary forest regenerating following shifting cultivation may be a major but uncertain source in the Philippines^30^. Jiang *et al*. estimated the current terrestrial carbon sink in China to be 0.16–0.35 PgC/yr. However, these estimates are subject to uncertainty because of the simplicity of the atmospheric networks in the past^31^.

There are many methods for assessing carbon sink functions. Carbon balance methods—for traditional, regional-scale terrestrial ecosystems—mainly consist of inventory investigations of plant biomass and soil carbon stores, ecosystem flux observations, satellite remote sensing surveys, atmospheric CO_2_ concentration inversions, and models to simulate ecological systems^32^. In this study, we applied the CASA model and photosynthetic reaction equation, and showed that plants act as carbon sinks and absorb CO_2_ through photosynthesis using water from the atmosphere^33^. Different carbon sink evaluation methods have specific advantages and disadvantages; however, evaluations at different scales of regional ecosystem carbon sinks are subject to large uncertainties^34, 35^. Piao *et al*. used process and atmospheric inversion models, and remote sensing methods to evaluate China’s terrestrial ecosystem carbon balance, finding that different methods applied to the same area (such as the northeast region) could give very different conclusions^36^.

Urbanization leads to significant changes in surface coverage; it is a complex process, with impacts on the regional ecosystem carbon cycle. The urban ecosystem carbon cycle has binary composite characteristics of both the natural ecosystem and social and economic systems^37^; this increases the uncertainty. These complexities of urban ecology thus merit further investigation.

## Method

### Study area

**Figure 3:**
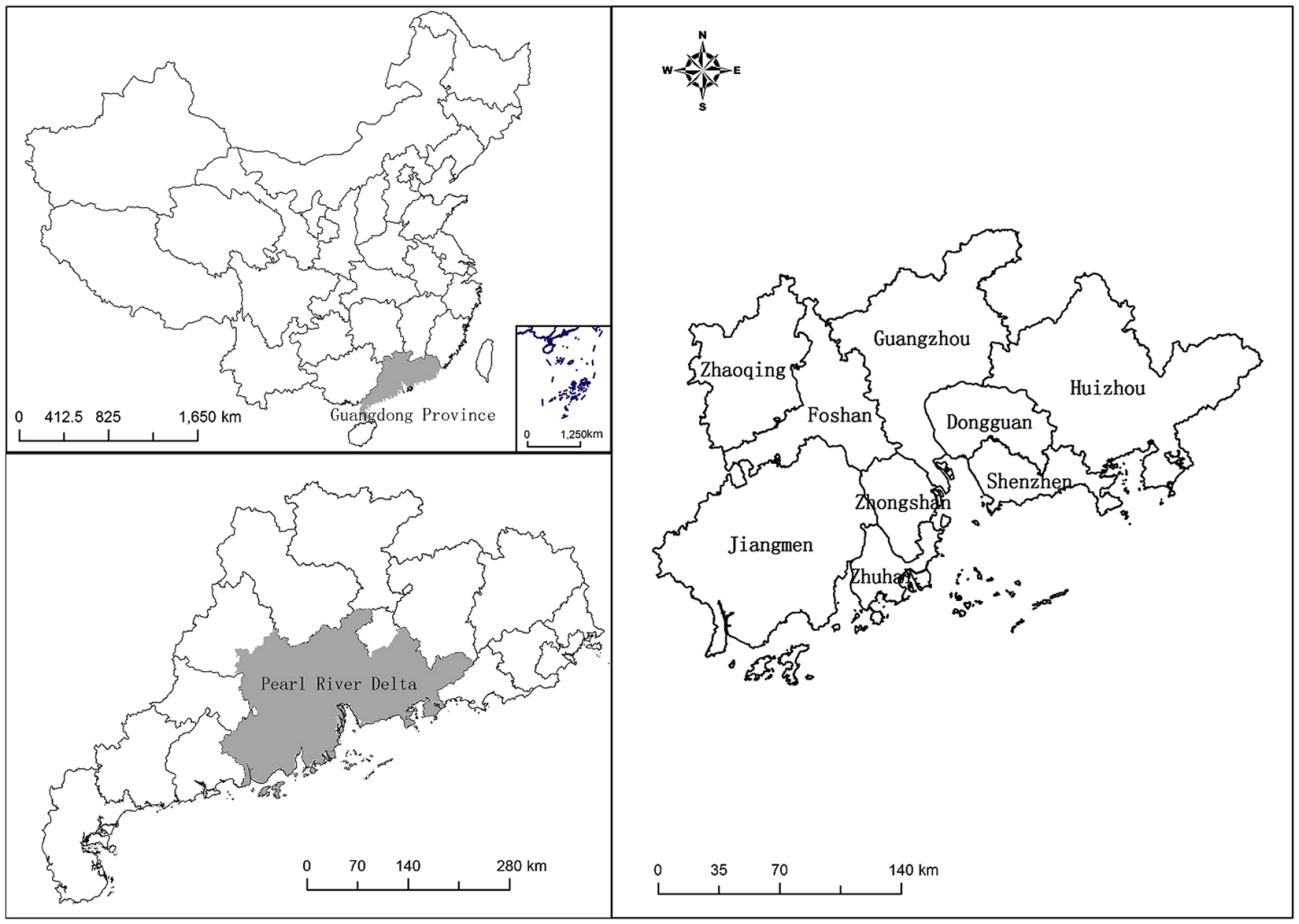
Location of the study area. Map created using ArcMap (version 10.2) software, (http://www.arcgis.com/). (Scientific Reports remains neutral with regard to jurisdictional claims in published maps).

The Pearl River Delta area (21°17.6′N–23°55.9′N, 111°59.7′E–115°25.3′E) is formed of alluvial deposits. It falls within the southern subtropics, with subtropical evergreen broad-leaved forest vegetation. Average rainfall is about 1600 mm, mainly concentrated in summer; conversely, winters are drier. The relief is flat and the area is bounded to the west and north by the Luoping Mountains. There are 1900–2200 sunshine hours per year, while the annual average daytime temperature is greater than 20 °C. Land use is characterized by circle-type development; the area of construction land has rapidly expanded, from 4137.69 km^2^ in 2000 to 7277.28 km^2^ in 2013, while areas of cultivated and forest land have declined rapidly. The land use structure is thus changing from complex to simple. In terms of administrative areas, Guangzhou city lies at the center of the Pearl River Delta, with Shenzhen, Foshan, and Zhuhai as sub-centers (Fig. 3). The Pearl River Delta encompasses an area of 41.09 × 10^3 ^km^2^, and has a permanent resident population of 57.15 million (2013), and urbanization rate with the proportion of urban residents being 84.03% (2013).

### Data collection

Land use data (from the years 2000, 2005, and 2013) were used for interpreting Landsat TM images. NDVI data from MODIS13Q1 were obtained via Earth Explorer, operated by the United States Geological Survey (http://earthexplorer.usgs.gov/); the data were pretreated, with atmospheric correction, radiation correction, and geometric correction. The spatial resolution was 250 m, while temporal resolution was 16 days. Data for total solar radiation and vegetation distribution were derived from the Data Sharing Infrastructure of Earth System Science (Chinese Academy of Sciences). Meteorological data (annual mean temperature, annual mean humidity, and annual rainfall) were obtained from a daily data set gathered from 25 benchmark ground meteorological stations and automatic stations within Guangdong province. These stations surround the study area, in accordance with the principle of spatial interpolation. Rasterized data layers for temperature and precipitation were obtained by interpolating a raster surface from points using Kriging; spatial resolution was 250 m, as for the NDVI data. Land use operations were used to derive the Landscape Diversity Index, Landscape Dominance Index, and Landscape Perimeter Area Fractal Dimension.

### CASA model

The study used the improved CASA (Carnegie–Ames–Stanford Approach) light energy utilization model to calculate net primary productivity (NPP). The model overcomes the disadvantages of using maximal light use efficiency (ε_max_) of world vegetation as a definite value. We referred to Zhu *et al*.^38^ for vegetation types and the characteristics of the regional natural environment in the Pearl River Delta area, in order to determine ε_max_ and for scientific evaluation of NPP quality in the Pearl River Delta area:

1 $${{\rm{NPP}}}_{year}=\,\sum _{i=1}^{12}APA{R}_{i}\times {\varepsilon }_{i}$$

where NPP_*year*_ is NPP for a particular year, *i* is month, *APAR* is Absorbed Photosynthetic Active Radiation, and *ε* is actual light energy utilization. Our NPP values were within a reasonable range of those given in other studies (Table 4).

**Table 4:** NPP values obtained in this study, compared with values from the literature.

Land use	NPP gC·m^−2^·a^−1^	Area	Method	Time	References
Average all types	829.63	Pearl River Delta	CASA	2000	This study
	753.2 (±277)	Guangdong Province	CASA	1992–1993	Guo^39^
	1480 (±407)	Guangdong Province	GLO-PEM	1981–2000	Liu^40^

In ecosystems, various vegetation types absorb carbon dioxide from the atmosphere by photosynthesis, releasing oxygen. The related chemical reaction is as follows:

2 $$6C{O}_{2}+6{H}_{2}O\to {C}_{6}{H}_{12}{O}_{6}+6{O}_{2}$$

Based on this photosynthetic reaction equation, the production of 1 kg of dry matter can fix 1.63 kgCO_2_^41^. Using the pre-measured amount of NPP material (the production of dry matter in an ecosystem) and the photosynthetic reaction equation, we calculated the fixed amount of CO_2_ per unit area of the ecosystem. Then, based on the coefficient 12/44 (the ratio of the relative molecular masses of carbon and CO_2_), we obtained the amount of fixed carbon. As noted, carbon sinks represent processes and mechanisms through which plants absorb CO_2_ and water from the atmosphere via photosynthesis, fixing CO_2_ in plants, with this being translated into carbon in soil^33^. For the purposes of this study, the amount of fixed carbon was used to approximate carbon sinks.

### GeogDetector

Using GeogDetector, we detected and identified geographical factors affecting the land use carbon sink function of the Pearl River Delta region^42, 43^:

3 $${{\rm{P}}}_{D,U}=1-\frac{1}{n{\sigma }_{u}^{2}}\sum _{i=1}^{m}{n}_{D,i}{\sigma }_{{U}_{D,i}}^{2}$$

where P_*D,U*_ is the power of the determinant, *n* is all sample numbers from the study area, ${\sigma }_{u}^{2}$ is the variance of the carbon sink, and (D,*i*) denotes a sub-area. The model was based on the hypothesis ${\sigma }_{{U}_{D{,}i}}^{2}\ne 0$, with ${P}_{D,U}\in [0,1].$ A significantly larger value of P_*D,U*_ represents greater power of influence of a factor, and consequently a higher degree of influence on the land use carbon sink.

**Figure 4:**
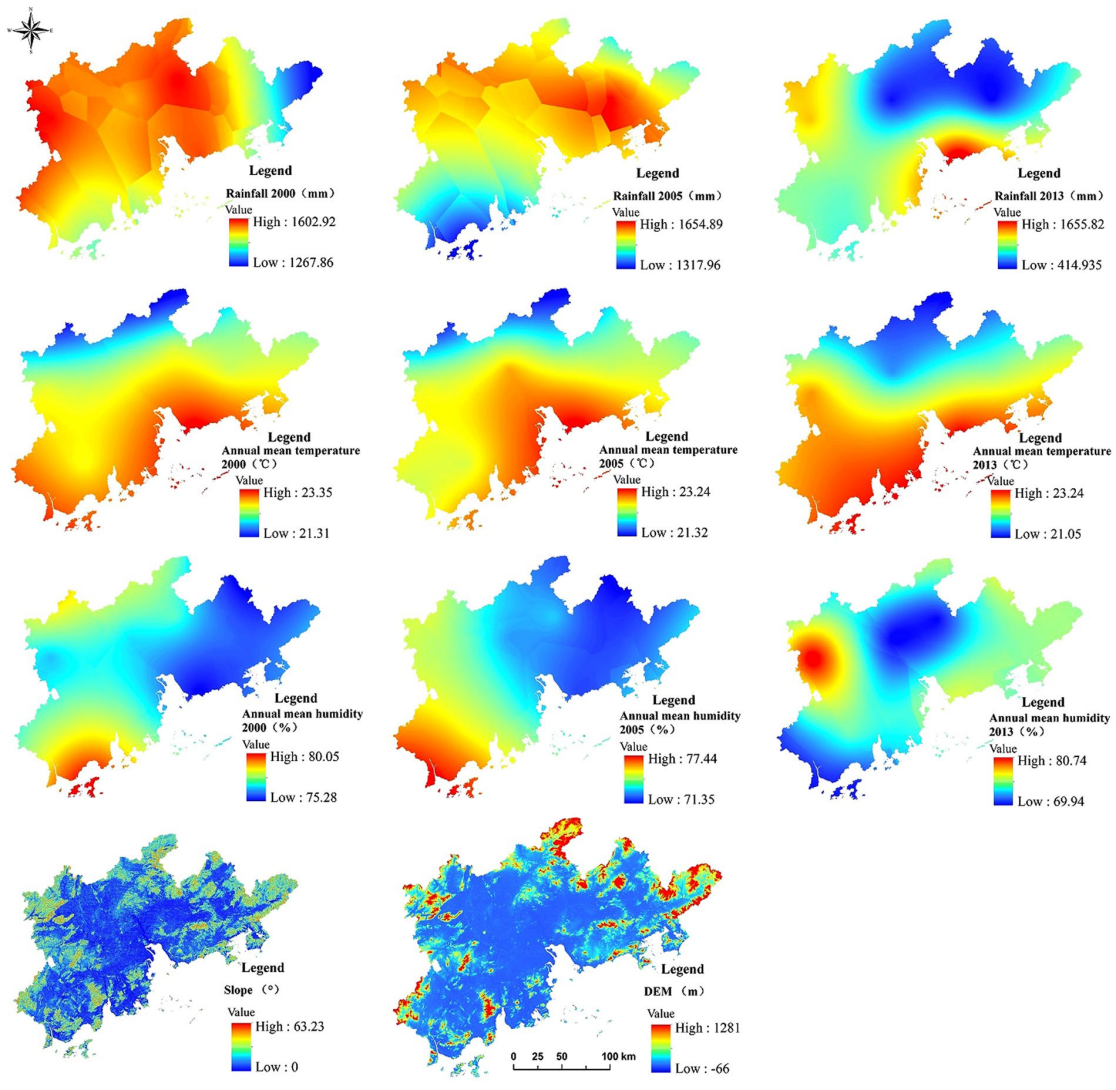
Natural conditions in the Pearl River Delta area. Map created using ArcMap (version 10.2) software from Esri (http://www.arcgis.com/).

The present study focuses on three different categories of natural conditions, the level of urbanization, and land use change. Eleven indices were selected to provide a comprehensive assessment of geographical factors affecting the carbon sink function in the Pearl River Delta. Natural factors mainly included regional climate, topography, and landform (Fig. 4), including annual mean temperature (f_1_), annual rainfall (f_2_), annual mean humidity (f_3_), altitude (f_4_), and slope (f_5_). George^44^ argued that a key feature of Chinese urbanism is the rapid growth in urban size. When measuring China’s urbanization level, land urbanization is more important than population urbanization; consequently, the present study adopted the urban land expansion scale perspective, with two related indices: urban land proportion f_6_ (the proportion of urban area within the total area of large, medium, and small cities and countryside) and the proportion of other construction land f_7_ (including industrial land, traffic land, and special land). Rapid urbanization and construction of urban infrastructure obviously influence the urban and landscape structures, leading to landscape fragmentation and inefficient use of the natural landscape^45^. Urbanization is a process in which natural landscapes are converted to artificial landscapes. The landscape of region is changed obviously. The characters of urban landscape are huge and rules artificial landscape to high concentration. The landscape diversity has been changed by urbanization. Simplification of landscape diversity greatly influence on landscape ecological evolution process of urban system^46, 47^. Therefore, the Landscape Diversity Index f_8_ (measuring the complexity of system structure), the Landscape Dominance Index f_9_ (with larger dominance index indicating that one or a few patch types dominate the landscape), and the Landscape Perimeter Area Fractal Dimension f_10_ (measuring adjacency between different patches of the same type, and landscape fragmentation) are selected. These landscape metrics can characterize the rapid urbanization, which has changed the regional landscape pattern and will affect land use structure and the carbon sink effect. These were combined with spatial pattern analysis of landscape ecology for a thorough analysis of the effect on carbon sinks of landscape space, the layout character of land use change, and related evolutionary processes. Using GeogDetector, all factors were divided into four levels (Table 5), with the altitude division standard being <10 m approximating sea level, <200 m indicating plains, and >500 m indicating mountains. The slope factor was subdivided into flat slope (0–6°), gentle slope (6–15°), steep hill (15–25°), and abrupt slope (25–90°). Land urbanization was categorized on the basis of the three stages of the urbanization process. The “*Report 2013 of China*’*s modernization - research of city modernization*”^48^ identified the following stages: (i) early urbanization, when urbanization level is <30% and urbanization speed is slow; (ii) an urbanization level of 30–70%; and (iii) late urbanization, when urbanization level is >70% and urbanization speed is slow. The rest of the factors were divided according to the same sample size. The rater data grid size of all impact factors should match that of the NDVI data, and data accuracy was thus 250 m. Using a 3 km × 3 km research scale for the Pearl River Delta area, the study area was subdivided into secondary areas. Using a geographical information system (GIS), the region was thus divided into 5150 grid units, with these used to analyze the relative strengths of different influence factors.

**Table 5:** Impact factor partitions for the identified geographical factors.

	f_1_ (°C)	f_2_ (mm)	f_3_ (%)	f_4_ (m)	f_5_ (°)	f_6_ (%)	f_7_ (%)	f_8_	f_9_	f_10_
First-grade regions	≤21.5	≤1050	≤75	≤10	≤6	≤30	≤0	≤0.35	≤0.5	≤0.8
Second-grade regions	(21.5,22.3)	(1050,1300)	(75,76)	(10,200)	(6,15)	(30,50)	(0,20)	(0.35,0.7)	(0.5,0.8)	(0.8,1.3)
Third-grade regions	(22.3,23)	(1300,1600)	(76,77)	(200,500)	(15,25)	(50,70)	(20,50)	(0.7,1)	(0.8,1.2)	(1.3,1.8)
Fourth-grade regions	>23	>1600	>77	>500	>25	>70	>50	>1	>1.2	>1.8
