# Erratum: Influence of different geographical factors on carbon sink functions in the Pearl River Delta

**DOI:** 10.1038/s41598-017-04856-6

**Published:** 2017-07-20

**Authors:** Qian Xu, Yuxiang Dong, Ren Yang

**Affiliations:** 1 Guangdong Provincial Key Laboratory of Urbanization and Geo-simulation, Centre of Land Research, School of Geography and Planning, Sun Yat-sen University, Guangzhou, 510275 PR China; 2 Xinhua College of Sun Yat-sen University, Guangzhou, 510520 PR China

*Scientific Reports* **7**:110; doi:10.1038/s41598-017-00158-z; Article published online 08 March 2017

In the original version of this Article, Yuxiang Dong was incorrectly listed as being affiliated with ‘Guangdong Provincial Key Laboratory of Urbanization and Geo-simulation, Centre of Land Research, School of Geography and Planning, Sun Yat-sen University, Guangzhou, 510062, PR China’. The correct affiliation is listed below:

‘Guangdong Provincial Key Laboratory of Urbanization and Geo-simulation, Centre of Land Research, School of Geography and Planning, Sun Yat-sen University, Guangzhou, 510275, PR China’.

